# The bifactor model of the Hungarian self-report version of the Strengths and Weaknesses of ADHD and Normal Behaviors scale

**DOI:** 10.1192/j.eurpsy.2024.201

**Published:** 2024-08-27

**Authors:** K. Vajsz, L. R. Paulina, M. Miklósi

**Affiliations:** ^1^Department of Clinical Psychology, Semmelweis University; ^2^Department of Developmental and Clinical Child Psychology, Eötvös Loránd University; ^3^Centre of Mental Health, Heim Pál National Pediatric Institute, Budapest, Hungary

## Abstract

**Introduction:**

Attention Deficit/Hyperactivity Disorder (ADHD) is one of the most common neuropsychiatric conditions, maintaining its presence well into adolescence and adulthood, resulting in impaired functioning. Evaluating ADHD symptoms through self-reporting plays a crucial role in assessing individuals within these age groups. The novel self-report version of the Strengths and Weaknesses of ADHD and Normal Behaviors (SWAN) scale offers a comprehensive assessment of behaviour, extending beyond just focusing on the typical signs and symptoms of ADHD, thus providing a more holistic perspective.

**Objectives:**

Our goal was to assess the factorial validity of the Hungarian version of the SWAN self-report by comparing a two-factor model with bifactor models with a general and 1) two specific factors (inattention, hyperactivity/impulsivity), 2) three specific factors (inattention, motor hyperactivity/impulsivity, verbal hyperactivity/impulsivity) in a community sample.

**Methods:**

Data from 717 adolescents and young adults (mean age = 20.0 years, SD = 3.10, range: 14 - 25 years, female: N = 664, 92.6%) were analysed. Participants completed an online questionnaire including the SWAN scale after giving informed consent. Confirmatory factor analyses were conducted based on the maximum likelihood estimator (ML).

**Results:**

The bifactor model with a general and three specific factors demonstrated the best fit to our data (CFI = .933, RMSEA = .064 [90% CI: .058 – .071], SRMR = .038). While the overall composite reliability was excellent (ω = .91), the reliability of the specific verbal hyperactivity/impulsivity factor fell below acceptable (ω_h_ = .40).

**Conclusions:**

In line with previous studies, the fit indices of the bifactor models were superior to the non-hierarchical two-factor model. Our results support the existence of a strong general factor but suggest uncertainty in the capacity of the specific factors to consistently explain the distinct variance in observed variables, particularly when compared to the overarching influence of the general factor.

This work was supported by the ÚNKP-22-2 New National Excellence Program of the Ministry for Culture and Innovation from the source of the National Research, Development and Innovation Fund (grant number ÚNKP-22-2-I-ELTE-854).

**Disclosure of Interest:**

None Declared

